# Beyond the basics: rethinking antimicrobial stewardship by targeting cytomegalovirus immune globulin for immunocompromised patients

**DOI:** 10.1017/ash.2025.10074

**Published:** 2025-07-31

**Authors:** Ritika Prasad, Brian Lu, Daniella Veloria, Emily Mui, Joanna Nelson, Gundeep Dhillon, Stanley Deresinski, Marisa Holubar, William Alegria

**Affiliations:** 1 Division of Infectious Diseases and Geographic Medicine, Department of Medicine, Stanford University School of Medicine, Stanford, CA, USA; 2 Department of Quality, Safety & Health Equity, Stanford University School of Medicine, Stanford, CA, USA; 3 Department of Pharmacy, Stanford Health Care, Stanford, CA, USA; 4 Division of Pulmonary, Allergy and Critical Care Medicine, Department of Medicine, Stanford University School of Medicine, Stanford, CA, USA

## Abstract

Identifying actionable stewardship targets in immunocompromised patients is challenging due to limited data and high morbidity. One approach could be targeting therapy with limited evidence, like cytomegalovirus immune globulin (CMV-IGIV). We implemented a drug restriction program that increased appropriate use of CMV-IGIV, highlighting a unique stewardship opportunity in immunocompromised populations.

## Introduction

Cytomegalovirus (CMV) infection causes significant morbidity and mortality in solid organ and hematopoietic stem cell transplant recipients. Although CMV immunoglobulin (CMV-IGIV) was first approved for prophylaxis by the Food and Drug Administration in 1987, advancements in diagnostics and therapeutics have since improved the management of CMV disease. Consequently, the role of CMV-IGIV is now unclear. Although guidelines suggest its possible role as an adjunct to antivirals for prevention of CMV infection in high-risk lung transplant or small bowel transplant recipients, or for treatment of refractory or resistant CMV disease, they acknowledge that this evidence base is limited, with no randomized trials assessing the additive impact of CMV-IGIV to antiviral therapy.^1^ Similarly, it is not clear that CMV-IGIV provides benefit over conventional immune globulin (IVIG); no high quality trials compare the efficacy of these therapies.

Furthermore, the high cost of CMV-IGIV should be considered. Since CMV-IGIV is commonly distributed in single-use vials, if a dose is ordered without rounding to the nearest vial size, there is a high potential for medication waste and avoidable expense.^2^ While CMV-IGIV has traditionally been dosed by actual body weight (ABW), work examining conventional IVIG has demonstrated that dosing by ideal body weight (IBW) instead of ABW does not lead to worse outcomes, suggesting an opportunity for cost savings by dosing CMV-IGIV by IBW.^2,3^

Recent publications have highlighted the role of antimicrobial stewardship programs (ASPs) in areas beyond antibiotic use, such as the design and implementation of delivery systems for monoclonal antibodies.^4,5^ Similarly, CMV-IGIV may be an attractive target for ASPs focused on interventions in transplant recipients, a population traditionally difficult to target for stewardship due to limited data and high risk of infection-related morbidity and mortality.^6^ We implemented a CMV-IGIV drug restriction policy outlining appropriate indications and dosing and evaluated its effect on prescribing practices and cost savings.

## Methods

In the CMV-IGIV restriction policy, we defined high-risk transplant patients as lung transplant patients with a positive donor CMV IgG and negative recipient CMV IgG serology. CMV-IGIV orders were categorized based on either prophylaxis or treatment of CMV. We defined appropriate indications for prophylaxis as: (1) High-risk lung transplant recipients or (2) other high-risk transplant patients meeting all the following criteria: intolerant of valganciclovir or ganciclovir, not receiving additional CMV therapy, and formal ID consultation. Appropriate treatment indications included use as an adjunct to antiviral therapy in transplant recipients with resistant or refractory CMV disease, or in those with serious clinical manifestations, as determined by the treating clinician. The drug restriction policy required dosing by IBW rather than ABW, with doses rounded to the nearest vial size (2.5 g).

In this quality improvement project, we reviewed adults ≥18 years who received inpatient and/or outpatient CMV-IGIV at Stanford Health Care before and after implementation of the CMV-IGIV restriction policy (prerestriction cohort: 09/01/2020–8/31/2022; postrestriction cohort: 9/1/2023–6/30/2024.) We compared the percentage of appropriate orders in both cohorts. We reported the number of patients who received CMV-IGIV for prophylaxis and subsequently developed CMV DNAemia (defined as above the lower limit of quantitation, 135 IU/ml). Using the current CMV-IGIV wholesale price of $2,108 per 2.5 g vial, we estimated cost savings by comparing the observed cost in the postrestriction cohort to the hypothetical cost if doses had been calculated by ABW without rounding to the nearest vial size.^7^

## Results

Of the 65 patients who received CMV-IGIV during the review period, 43 were in the prerestriction cohort and 22 were in the postrestriction cohort (Table 1). A total of 261 CMV-IGIV orders were placed (prerestriction: 168 orders, postrestriction: 93 orders; Table 2). High-risk transplant recipients accounted for the majority of CMV-IGIV use in both cohorts (prerestriction cohort: 65% [28/43]; postrestriction cohort: 91% [20/22]; Table 1). In the prerestriction cohort, we observed that CMV-IGIV was used in a heterogeneous group of patients with a variety of transplant types and other immunocompromising conditions; after drug restriction, CMV-IGIV use was only seen in lung transplant patients (Table 1). Receipt of conventional IVIG in the 30 days prior to CMV-IGIV use occurred in 7% (3/43) of patients in the prerestriction cohort and 41% (9/22) of patients in the postrestriction cohort (Table 1). Postrestriction IVIG was given for non-CMV related reasons (e.g. hypogammaglobulinemia or prevention of transplant rejection).

**Table 1.** Patient characteristics of pre and postrestriction cohorts

	Prerestriction (n = 43)	Postrestriction (n = 22)
Median age, years (IQR)	62 (45–62)	63 (54–69)
Female sex, n (%)	12 (28%)	8 (36%)
Transplant type, n (%)		
Lung	32 (74%)	19 (86%)
Heart/Lung	4 (9%)	2 (9%)
Liver/Lung	0 (0%)	1 (5%)
Kidney/Lung	1 (2%)	0 (0%)
Pancreas/Kidney	1 (2%)	0 (0%)
Liver	3 (7%)	0 (0%)
Hematopoietic stem cell transplant	1 (2%)	0 (0%)
None	1 (2%)	0 (0%)
CMV risk category, n (%)		
High risk (D+/R-)	28 (65%)	20 (91%)
Intermediate risk (D-/R+, D+/R+)	12 (28%)	2 (9%)
Low risk (D-/R-)	2 (5%)	0 (0%)
Not applicable	1 (2%)	0 (0%)
CMV-IGIV indication, n (%)		
Prophylaxis	27 (63%)	21 (95%)
Treatment	16 (37%)	1 (5%)
Received IVIG within 30 days prior to CMV-IGIV, n (%)	3 (7%)	9 (41%)

**Table 2.** Appropriateness of CMV-IGIV orders before and after implementation of drug restriction policy^a^

Indication		Prerestriction (n = 168)		Postrestriction (n = 93)	
Prophylaxis orders, n (%)		146 (86%)		92 (99%)	
Appropriate indications	High risk lung transplant, n (%)	131 (90%)	Total131 (90%)	89 (97%)	Total89 (97%)
	Other high-risk transplant intolerant of GCV^b^/VGCV^c^, not on CMV antiviral, and ID consult, n (%)	0 (0%)		0 (0%)	
Inappropriate indications	Intermediate risk transplant, n (%)	1 (1%)	Total15 (10%)	1 (1%)	Total3 (3%)
	Posttransplant, intolerant of GCV^b^/VGCV^c^, and no ID consult, n (%)	14 (10%)		2 (2%)	
Treatment orders, n (%)		22 (13%)		1 (1%)	
Appropriate indications	Refractory or resistant CMV disease, n (%)	5 (23%)	Total11 (50%)	1 (100%)	Total1 (100%)
	Serious clinical manifestation, n (%)	6 (27%)		0 (0%)	
Inappropriate indications	Asymptomatic CMV DNAemia, n (%)	10 (45%)	Total11 (50%)	0 (0%)	Total0 (0%)
	Asymptomatic CMV detected in bronchoalveolar lavage, n (%)	1 (5%)		0 (0%)	

a Percentages may not total to 100% due to rounding.

b GCV = Ganciclovir.

c VGCV = Valganciclovir.

We observed a reduction of CMV-IGIV treatment orders after drug restriction (prerestriction: 13% [22/168], postrestriction: 1% [1/93]; Table 2), with most postrestriction use limited to prophylaxis. The appropriateness of CMV-IGIV treatment orders rose from 50% [11/22] prerestriction to 100% [1/1] postrestriction, largely by eliminating use for asymptomatic CMV DNAemia (Table 2). Among those who received CMV-IGIV for prophylaxis, 1 patient in both the pre and postrestriction cohorts developed CMV DNAemia (Table 1).

We observed that dosing based on IBW and rounding to the nearest vial size led to significant cost savings. The cost of all CMV-IGIV orders postrestriction was $554,404. Had this cohort been dosed by ABW without rounding to vial size, the wholesale cost would have been $723,044.

## Discussion

After implementing a drug restriction policy, we observed a shift in prescribing that ultimately culminated in more standardized utilization. Our experience targeting a niche polyclonal antibody used almost exclusively in immunocompromised patients highlights an opportunity for stewardship programs looking to pursue similar interventions. Through critical appraisal of the literature, development of drug use criteria, and multidisciplinary collaboration, stewardship teams can impact transplant populations and curtail unnecessary drug expenditure.

We found that several patients in both the pre and postrestriction cohorts received conventional IVIG within 30 days prior to CMV-IGIV, a potentially redundant practice given that the half-life of conventional IVIG is 3–4 weeks and it may have some activity against CMV.^8^ Although *in vitro* studies have demonstrated that CMV-IGIV products have higher CMV antibody titers than conventional IVIG products, there are mixed data comparing neutralizing activity of CMV-IGIV to conventional IVIG, and no randomized controlled trials in humans.^9,10^ Although our drug restriction policy did not restrict CMV-IGIV based on receipt of recent conventional IVIG, this could be a next step in reducing unnecessary CMV-IGIV use. We observed a higher proportion of IVIG use postrestriction, however, given the small number of patients receiving IVIG, it is difficult to interpret this trend. Additionally, these patients received IVIG for only non-CMV related reasons, suggesting IVIG usage changes were unrelated to the CMV-IGIV restriction policy.

This project has several limitations. First, it was observational, limiting our ability to infer causality between the drug restriction policy and improved prescribing practices. Other institutional changes or external factors may have influenced CMV-IGIV use. Second, our analysis did not assess clinical outcomes, such as rates of CMV disease or patient survival. Third, our single-center experience may have limited generalizability. Finally, we could not match pre and post time periods because shortly after completion of this project, CMV-IGIV was removed from formulary and the lung transplant prophylaxis protocol following discussions with our ASP, lung transplant and infectious diseases groups.

We demonstrated that ASPs can effectively implement drug restriction policies to improve appropriate prescribing and reduce expenditures in immunocompromised populations. Given the challenges in identifying stewardship targets for immunocompromised patients, our approach provides a model for similar interventions in other institutions. Continued efforts are needed to identify new stewardship opportunities in immunocompromised populations.
